# Beyond the Pandemic: Mental Health and Eight Dimensions of Wellness Among Nurses in Two Regional Hospitals in Albania

**DOI:** 10.3390/healthcare13161973

**Published:** 2025-08-11

**Authors:** Rudina Çerçizaj, Fatjona Kamberi, Emirjona Kiçaj, Vasilika Prifti, Sonila Qirko, Liliana Rogozea

**Affiliations:** 1 Faculty of Medicine, Transylvania University, 500036 Brasov, Romania; fatjona.kamberi@univlora.edu.al (F.K.); emirjona.kicaj@unitbv.ro (E.K.); vasilika.prifti@unitbv.ro (V.P.); sonila.qirko@unitbv.ro (S.Q.); r_liliana@unitbv.ro (L.R.); 2 Faculty of Health, University “Ismail Qemali” Vlore, 9401 Vlore, Albania

**Keywords:** nurses, mental health, COVID-19, well-being, depression, anxiety, stress, post-pandemic, Albania

## Abstract

**Background:** The COVID-19 pandemic has had a profound impact on nurses’ mental health and overall wellness, both during and after the crisis. **Objectives:** This study aims to explore overall wellness during and after the pandemic, and the long-term psychological effects of the relationship between psychological distress and the eight dimensions of well-being among nurses in the post-pandemic era. **Methods:** A cross-sectional research design was used to conduct the study among 288 nurses from two regional hospitals in Albania. Participants were recruited using purposive sampling, selecting nurses based on availability and relevance to the study criteria. Data were collected through self-administered questionnaires using the DASS-21 scale and the Personal Assessment of the Eight Dimensions of Wellness. Descriptive statistics and Kendall’s Tau-b correlation were used to assess associations, followed by ordinal regression to explore the influence of demographic and professional variables. **Results:** Findings revealed persistent levels of psychological distress among nurses, especially related to anxiety and stress. Significant negative correlations were found between wellness dimensions particularly emotional and occupational wellness and psychological distress. Age and department assignment emerged as predictors of post-pandemic stress and depression. **Conclusions:** The study highlights the need for institutional strategies to support mental health and promote comprehensive well-being among nurses in the post-COVID-19 period. Investing in long-term psychological support and wellness training is essential for building a resilient nursing workforce.

## 1. Introduction

The COVID-19 pandemic had a profound and lasting impact on global healthcare systems, placing extraordinary demands on healthcare professionals, especially nurses. Being at the forefront of the response, nurses endured intense physical and emotional strain, often working under extreme conditions marked by isolation, personal loss, and a severe lack of protective resources [1,2]. These experiences have left a psychological toll that, for many, did not end when the crisis did.

Although much of the early research has explored the immediate mental health effects of the pandemic on nurses, a notable gap remains in understanding how these impacts have evolved [3,4]. Growing evidence indicates that conditions such as anxiety, depression, and chronic stress may persist long after the peak of the pandemic, influencing not only nurses’ personal lives but also their ability to perform professionally.

To fully grasp the post-pandemic challenges faced by nurses, it is essential to move beyond symptom-based assessments. The eight-dimensional model of wellness, which encompasses emotional, physical, social, spiritual, intellectual, environmental, financial, and occupational well-being, offers a broader and more integrative view of health and recovery [5,6].

Existing studies highlight that some wellness dimensions, particularly emotional, spiritual, and occupational have been severely affected during the pandemic and may also serve as key buffers in maintaining mental health [7,8,9,10]. Supporting these areas may enhance nurses’ resilience and their capacity to cope with ongoing stressors in the healthcare environment.

In the Albanian context, the long-term mental health and wellness of nurses remain largely understudied. With limited structured support and few targeted interventions, this population has received little sustained attention, despite playing a critical role during the pandemic. In Albanian hospitals, for example, nurses reported lacking not only psychological support but also structured debriefing after each pandemic wave. Addressing this gap is crucial for informing national strategies that aim to enhance psychological support and promote well-being among nurses in the post-COVID-19 era.

Our study aims to assess the long-term impact of the COVID-19 pandemic on the mental health of nurses, analyzing levels of depression, anxiety, and stress during and after the pandemic, and to assess their well-being in the post-pandemic period. Furthermore, the study examines the relationship between mental health and each dimension of well-being to identify key areas that can serve as intervention points for improving nurses’ professional and personal well-being in the future. We chose to base our study on Hettler’s model of well-being, which sees health as a balance between eight essential aspects of life. This approach allowed us to think more broadly about the long-term effects of the pandemic not only on nurses’ mental health but also on how they function and feel at work, in their families, and in their personal development. Based on this approach, we formulated the following research questions:

1. What was the impact of the COVID-19 pandemic on the mental health of nurses, analyzing the levels of depression, anxiety, and stress during and after the pandemic?
2. How does the well-being of nurses appear in the post-pandemic period, according to the eight dimensions of Hettler’s model?
3. Is there a statistically significant relationship between depression, anxiety, and stress and general well-being in the eight dimensions?
4. What demographic and professional factors predict the levels of mental distress and well-being of nurses after the pandemic?

## 2. Materials and Methods

### 2.1. Study Design

This study employed a cross-sectional research design using a mixed-method approach (quantitative and qualitative), aiming to assess the long-term impact of the COVID-19 pandemic on the mental health and well-being of nurses, based on the perceptions of those who worked during the pandemic.

### 2.2. Study Settings

The research took place between March and April 2024 in the regional hospitals in southern Albania: Fier Regional Hospital and Vlora Regional Hospital. These hospitals were purposively selected because they served as major healthcare centers during the pandemic, and their nursing staff directly faced the challenges of COVID-19. The study commenced immediately after obtaining ethical approval on 13 March 2024, and was conducted from 20 March to 10 April 2024.

### 2.3. Study Population and Samling

The study population included only nurses employed at Fier and Vlora Regional Hospitals, who provided care during the COVID-19 pandemic and continue to be active in the profession. Nurses from primary healthcare centers or other healthcare institutions, as well as nursing students and volunteers, were excluded from this study. Additionally, nurses who were not working during the pandemic period were not eligible to participate.

Inclusion criteria required participants to have been employed in hospital settings in Fier and Vlora throughout the pandemic period, with direct involvement in patient care. This was to ensure that the sample reflected the experiences of healthcare professionals who were directly exposed to the challenges and demands of the COVID-19 health crisis.

#### Study Sample

The total population of nurses employed at Fier and Vlora Regional Hospital during the study period was 505 (260 in Vlora and Fier). To determine the minimum required sample size, Fisher’s formula for finite populations was applied, assuming a 95% confidence level, a 5% margin of error, and an expected proportion (*p*) of 0.5 to maximize sample size.

The calculation yielded a minimum sample size of approximately 219 participants to ensure sufficient statistical power and representativeness of the target population.

A total of 300 questionnaires were distributed, and 288 completed responses were received, exceeding the minimum required sample size. The higher number of participants improves the precision of the estimates and increases the generalizability of the findings within the study population. Additionally, collecting more responses helps to mitigate the potential effects of incomplete data or non-response bias.

Sampling was achieved through purposive selection, including nurses with direct experience in treating patients during the pandemic who were available to participate in the study. This approach was appropriate for the exploratory context of the study; however, it may have created a selection bias due to the non-random nature of inclusion.

### 2.4. Data Sources/Measurement

The data were collected through structured questionnaires consisting of the following components:

- Socio-demographic data: includes basic data such as age, gender, education, professional experience, and place of work.
- DASS-21—Depression, Anxiety, and Stress Scale [11]: This is used to assess symptoms of depression, anxiety, and stress. This instrument has been translated and adapted into Albanian and underwent pilot testing with a small group of nurses before its release. Its use is free for research purposes.
- Personal Assessment: 8 Dimensions of Well-being [12]: This comprehensive questionnaire assesses personal well-being holistically in eight dimensions. Emotional well-being: the ability to manage emotions and stress while maintaining a positive outlook. Spiritual well-being: the search for meaning and purpose in life. Physical health: caring for the body through nutrition, physical activity, sleep, and avoidance of harmful substances. Social well-being: building healthy relationships and a sense of belonging in the community. Financial well-being: the ability to manage finances effectively and plan for the future. Professional well-being: fulfillment and satisfaction at work, maintaining a balance between personal and professional life. Intellectual well-being: engaging in activities that stimulate critical thinking, creativity, and lifelong learning. Environmental well-being: the individual’s relationship with the environment that surrounds them, including sustainability and safety.
- In this study, we utilized two validated tools to gain a deeper understanding of the mental health and overall well-being of nurses in the post-pandemic period: the DASS-21 (Depression, Anxiety, and Stress Scale) and Personal Assessment: 8 Dimensions of Wellness.

Both instruments were carefully translated into Albanian through a forward and backward translation process, ensuring that the meaning and intent of each item were accurately preserved. The adapted versions were then reviewed by professionals in mental health, nursing, and public health to ensure cultural and linguistic appropriateness for our context. This process was guided by Beaton et al.’s (2000) guidelines for cross-cultural adaptation of measurement instruments [13], ensuring semantic, conceptual, and cultural equivalence. This study is based on Hettler’s theoretical model of well-being, which divides well-being into eight interrelated dimensions: emotional, physical, social, spiritual, intellectual, professional, financial, and environmental. This theoretical framework has guided the analysis of the long-term impact of the pandemic, viewing well-being [14], as a complex and holistic construct that realistically reflects the challenges nurses face in all aspects of life.

Before proceeding with the comprehensive study, we conducted a pilot test to ensure the questions were clear, relatable, and easy to complete. Based on participant feedback, minor adjustments were made to improve clarity.

To assess internal reliability, we calculated Cronbach’s Alpha for each instrument. The DASS-21 showed strong internal consistency (α = 0.82), supporting its reliability in capturing emotional distress. The Wellness Assessment also demonstrated solid reliability (α = 0.85), confirming its value in reflecting the multidimensional nature of well-being.

Although the instruments are not specifically focused on the pandemic, participants were clearly instructed to answer the questions by reflecting on their experiences during and after the COVID-19 pandemic. This thoughtful adaptation process allowed us to ensure that the tools were not only statistically sound but also meaningful and respectful of the lived experiences of our participants.

#### 2.4.1. Questionnaire Distribution and Data Collection

Questionnaires were distributed among nurses working at the Fier and Vlora Regional Hospitals. The distribution targeted nurses who were actively working during the COVID-19 pandemic and who were present at the workplace at the time of data collection. A purposive sampling approach was used to identify eligible participants based on their direct involvement in patient care during the pandemic, followed by random selection of those available during the survey period.

Data collection was conducted in person, with researchers or designated coordinators providing the questionnaires directly to the participants. Completed questionnaires were collected immediately after completion or within a short period to ensure high response rates. Out of the 300 questionnaires distributed, 288 fully completed responses were returned and included in the analysis, representing a high participant rate of 96%.

#### 2.4.2. Pilot Study

Prior to the main data collection, a pilot study was conducted with 10 nurses from the same hospitals (not included in the final sample) to test the clarity, comprehension, and completion time of questionnaires. Minor adjustments were made based on feedback to ensure data quality.

Data were collected through paper-based questionnaires distributed and completed anonymously by nurses during their shifts. Hospital nursing supervisors assisted in organizing the distribution and collection process. Completed questionnaires were placed in sealed boxes to guarantee confidentiality.

### 2.5. Statistical Methods

In this study, data were gathered using two recognized questionnaires, both designed to explore different aspects of participants’ mental health and well-being.

#### 2.5.1. Measured Variables

Depression Anxiety Stress Scale-21 Items (DASS-21): this instrument measures emotional states across three areas: depression, anxiety, and stress.

Each of three subscales includes 7 items, and participants responded using a 4-point Liker scale from “Did not apply to me at all” to “Applied to me very much or most of the time.” To calculate the final scores, the responses in each subscale were summed and then multiplied by two. These scores were considered continuous variables, allowing us to analyze the intensity of psychological symptoms experienced by each participant.

Personal Assessment: 8 Dimensions of Wellness: this self-assessment tool offers a holistic perspective by exploring eight key dimensions of wellness: emotional, environmental, financial, intellectual, occupational, physical, social, and spiritual. Participants were asked to reflect on their current state in each dimension, and each area was treated as a separate, scalable 21-item scale during analysis. The tool encouraged participants to think about balance and well-being across different aspects of their lives.

#### 2.5.2. Data Analysis Procedures

All responses were initially entered in Microsoft Excel and then transferred to SPSS version 26.0., where the full data analysis was carried out.

To describe the characteristics of participants and their responses, categorical variables (such as gender or work setting) were summarized using frequencies and percentages.

Numerical variables (such as age or wellness scores) were summarized using means and standard deviations when the data followed a normal distribution.

When the data did not follow a normal distribution, we reported the median and the interquartile range instead.

The normality of the numerical data was tested using the Shapiro–Wilk test, which helped us decide which statistical measures were most appropriate.

To better understand the relationships between different aspects of mental health (from DASS-21) and the wellness dimensions, we used Kendall’s Tau-b correlation coefficient. This method allowed us to explore how variables moved together in meaningful ways. To analyze the impact of demographic and occupational factors on depression and anxiety levels after the pandemic, an ordinal logistic regression model was used. This model enables the analysis of ordered variables, such as depression and anxiety levels (categorized according to the DASS-21 scale), to assess whether factors like age, gender, work experience, training during COVID-19, and service in a COVID unit have a statistically significant impact on them.

For each model, the regression coefficients (B), standard errors (SE), Wald values, *p*-values, odds ratios (OR = Exp(B)), and 95% confidence intervals (95% CI) were reported.

The stress model was not included in the analysis due to lack of variation. Most participants reported normal stress levels in the post-pandemic period, making it impossible to build a valid model.

All statistical tests were considered significant if the *p*-value was less than 0.05, meaning there was less than a 5% probability that the findings were due to chance.

### 2.6. Ethical Considerations

The study received approval from the Research Ethics Committee at the Faculty of Health, University of Vlore “Ismail Qemali,” Vlore, Albania, under Decision No. 80, dated 13 March 2024. All participants provided informed consent and were assured of anonymity and confidentiality. Data were used solely for research purposes, adhering to ethical guidelines for human subject research.

All participants provided informed consent and were assured of anonymity and confidentiality. Data were used solely for research purposes, adhering to ethical guidelines for human subject research.

## 3. Results

A total of 300 questionnaires were distributed as part of the study. Of these, 288 were fully completed and met the inclusion criteria, providing complete and accurate data for analysis.

### 3.1. Socio-Demographic Data of Study Participants

Table 1 presents the descriptive distribution of demographic/occupational data, expressed in frequencies and percentages. Among the 288 nurses included in the study, 82.1% were female, while 17.9% were male. Most nurses reported being engaged in the frontline during COVID-19 (19.1%) and had 6–10 years of work experience (28.5%). Regarding the department in which they were engaged after COVID-19, the data are distributed as in Table 1. Both during and after the pandemic period, nurses reported working mostly 40 h a week, while 34%of them worked extended hours during the pandemic, and 26.4% continue to work over 40 h currently. When asked about training undertaken during COVID-19, 87.8% of nurses reported having received training.

### 3.2. Long-Term Impact of COVID-19 on Nurses’ Mental Health and Well-Being

Table 2 shows the depression indicators for numerical data (age, DASS during and after the pandemic, and the 8 Dimensions of Well-being). The study included 288 nurses, aged 22 to 62. Their mean age (±SD) was 38.6 ±10.4 years. The survey assessed the nurses’ levels of anxiety, stress, and depression during and after the COVID-19 pandemic. During the pandemic, nurses reported a higher level of depression (average 6.2 points) and anxiety (average 6.1 points), and stress was lower (average 8.3 points) compared to after the pandemic, when the levels of depression, anxiety, and stress are lower, with average values of 3.3 points, 2.9 points, and 4.8 points, respectively.

Regarding the assessment of well-being among nurses according to the eight dimensions, it resulted that their mean values (±SD) are 32.41 ± 6.44 for emotional well-being, 31.89 ± 6.55for spiritual well-being, 30.79 ± 5.77 for physical well-being, 32.32 ± 6.48 for social well-being, 31.36 ± 6.56 for financial well-being, 31.83 ± 6.68 for professional well-being, 32.17 ± 6.92 for intellectual well-being, and 32.18 ± 6.92 for environmental well-being.

The scores collected from the DASS questionnaires during and after the pandemic were categorised into severity levels of each psychological disorder (anxiety, stress, and depression) based on the cut-offs presented in Table 3, and the corresponding distribution is summarised in (Table 4).

As can be seen in Table 4, the majority of study participants did not experience mental health problems (depression, anxiety, orstress) either during or after the pandemic, specifically 84.4%during and 87.2% after for depression and 94.1% duringand95.5% after the pandemic for stress.

This study found that depression, anxiety, and stress were more prevalent during the COVID-19 period compared to the post-pandemic period.

Regarding depression levels, it is evident that the majority of nurses correspond to mild levels of depression for both periods, specifically 8% and 5.6%, followed by moderate levels of depression at 4.5% and 3.8% and severe levels at 3.5% and 3.1%.

**Figure 1:**
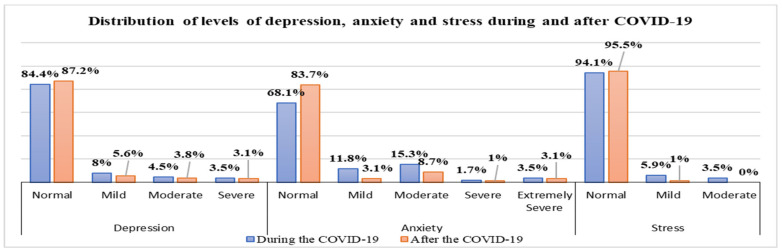
Distribution of levels of depression, anxiety, and stress during and after COVID-19.

Anxiety results as one of the most frequent psychological disorders encountered during the pandemic, compared to stress and depression, reaching the highest level of severity (extremely severe in 3.5%). Most of the nurses reported having experienced moderate levels of anxiety during the pandemic (15.3%), followed by mild levels (11.8%), while about 1.7% of the nurses experienced severe levels of anxiety. This study has identified stress at two levels: mild, occurring at a rate of 5.9% during the pandemic and 1% after, and moderate, at 3.5% during the pandemic. These data indicate that following the pandemic, there has been a significant improvement in the psychological state of nurses, with an increase in the percentage of those reporting normal levels of mental health. However, anxiety during the pandemic turns out to have been particularly widespread and with higher levels of severity compared to stress and depression, which indicates the need for emotional support for staff during health crises. Figure 1 reflects these data.

We explored how depression, anxiety, and stress relate to the eight dimensions of well-being by applying Kendall’s Tau correlation method. The results are presented in Table 5. The analysis shows that depression, anxiety, and stress have statistically significant negative relationships with the eight dimensions of well-being in the post-pandemic period, with a *p*-value of less than *p*< 0.05. The strongest negative associations were identified between spiritual, social, intellectual, and environmental well-being, particularly regarding depression and anxiety. These findings suggest that increased social belonging, spiritual development, and scholarly engagement may have a protective effect against psychological distress. Therefore, promoting multidimensional well-being should be an integral part of institutional support strategies for nurses in the post-pandemic period.

To assess the impact of demographic and occupational factors on the mental health of nurses following the pandemic, ordinal logistic regression was applied, with the results presented in the Table 6 below.

From the ordinal regression analysis between age, gender, work unit during COVID-19, work experience, COVID-19 training, and levels of depression, it was concluded that age was a positive factor (B = 0.084, *p* = 0.018), where with increasing age, the odds of experiencing higher levels of depression increase. Specifically, for each additional year of age, the odds of reporting a higher level of depression increase by 8.8% (OR = 1.088; 95% CI: 1.015–1.167).

Nurses who had worked in COVID-19 units during the pandemic were also significantly more likely to report higher levels of depression compared to their counterparts in other departments (B = 0.984, *p* = 0.020). The odds ratio (OR = 0.374; 95% CI: 0.163–0.857) suggests that working in COVID-19 units is associated with significantly higher odds of depressive symptoms, compared to other wards, reflecting the impact of exposure to high-stress environments during the pandemic. Professional experience results in a significant impact on post-COVID depression levels. Nurses with more than 10 years of work experience were over 16 times more likely to experience depressive symptoms (OR = 16.607; 95% CI: 1.775–155.336; B = 2.810, *p* = 0.014).

In contrast, gender and participation in COVID-19 training programs were not statistically significant predictors of depression levels after the pandemic (*p* > 0.05), indicating that these factors did not significantly contribute to the variation in depression levels among the study population.

Regarding the anxiety disorder encountered after the pandemic, in Table 7, variables that have an impact on its level are evaluated. The regression analysis results indicate that age is a statistically significant factor (B = 0.075, *p* = 0.025), suggesting that with each one-year increase in age, the likelihood of experiencing a higher level of anxiety increases by 7.8%. This indicates that older nurses have a higher probability (OR = 1.078) of the long-term effects of pandemic stress. Gender was still significant (*p* = 0.025), with male nurses being 61.5% less likely to report high levels of anxiety compared to females (OR = 0.385).

Nurses who had received training during the pandemic were less likely to experience anxiety (OR = 0.470); however, this was not statistically significant (*p* = 0.243). This suggests that training may have a protective role, but more evidence is needed.

Nurses with 11–15 years of experience were over 10 times more likely to experience anxiety compared to those with more than 15 years of work experience, which was used as a reference group. (OR = 10.11, *p* = 0.007).

Although this result may seem paradoxical at first glance, it can be explained by the fact that nurses working in COVID-19 wards had more clinical experience, support from colleagues, and more developed stress-coping strategies (OR = 0.240, *p* = 0.003). These elements may have positively influenced their psychological resilience, helping them to better cope with emotional challenges in difficult working conditions.

Post-pandemic stress was an essential component of the study, but it was not included in the advanced statistical analyses due to the lack of variance in the data. Since almost all participants reported normal levels (Figure 1), this did not allow the construction of a valid regression model. However, this finding is significant in itself and suggests a general improvement in the participants’ psychological well-being during the post-pandemic period.

### 3.3. Visual Presentation of Results

The scatter plots below graphically illustrate these results.

**Figure 2:**
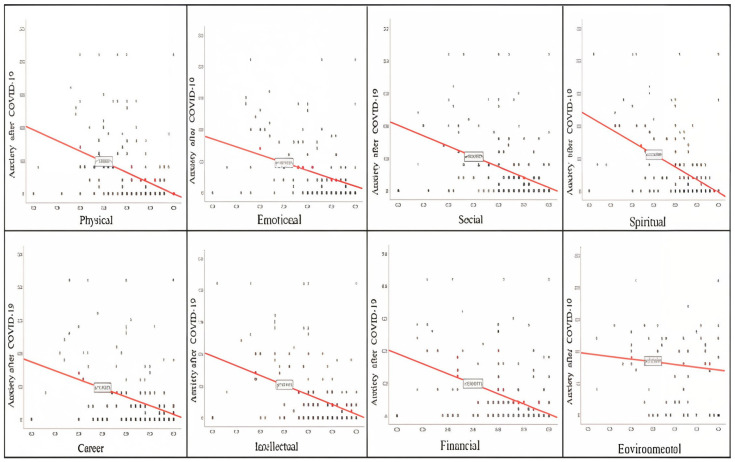
Relation between post-COVID-19 anxiety and dimensions of wellness.

Nurses with higher depression scores tended to report lower scores inemotional, intellectual, and spiritual wellness (Figure 2). This suggests a clear inverse relationship, consistent with the correlation results shown in Table 5.

**Figure 3:**
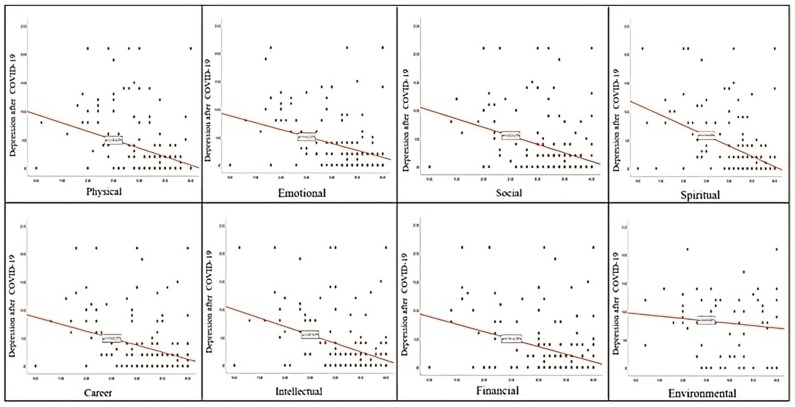
Relation between post-COVID-19 depression and dimensions of wellness.

A visible inverse trend is observed between anxiety levels and social/emotional wellness, highlighting how anxiety impacts interpersonal and emotional functioning (Figure 3).

**Figure 4:**
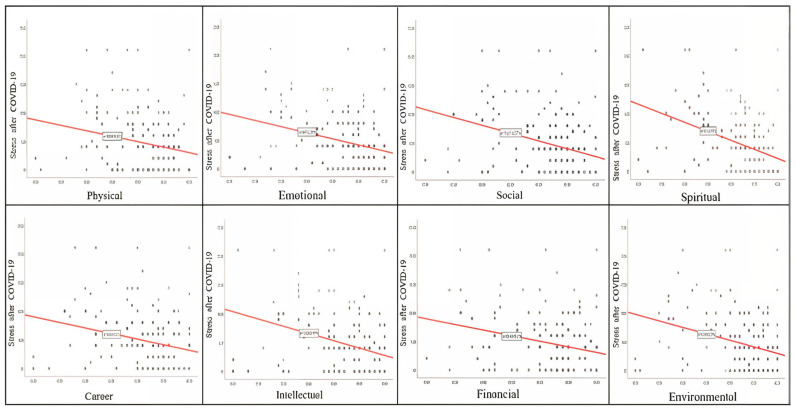
Relation between stress post-COVID-19 stress and dimensions of wellness.

Post-pandemic stress appears to be moderately negatively related to most wellness dimensions, primarily environmental and occupational wellness, reflecting lingering effects on the workplace (Figure 4).

## 4. Discussion

This study aimed to evaluate the long-term effects of the COVID-19 pandemic on the mental health of nurses, analyzing levels of depression, anxiety, and stress during and after the pandemic, as well as their well-being in the post-pandemic period through the eight dimensions of well-being.

In terms of understanding how the pandemic has affected nurses’ mental health over the long term, as frontline professionals during the global health crisis, nurses have carried a heavy emotional, physical, and social burden that continues to shape their daily lives.

While the acute crisis of the pandemic has subsided, its psychological and social consequences continue to affect nurses’ professional and personal functioning. A comprehensive assessment that includes not only mental health but also well-being in all its emotional, physical, social, spiritual, intellectual, environmental, financial, and professional dimensions provides complete pictures of their real needs and helps to design sustainable development of this profession.

The results show a significant difference between the periods before and after the pandemic in all three assessed components of mental health: depression, anxiety, and stress. Specifically, the mean of depression was 6.22 during the pandemic and 3.26 after it; anxiety fell from 6.11 to 2.91, while stress fell from 8.3 to 4.75. This reduction reflects the health emergency crisis and the gradual return to a more stable routine in nursing practice.

Although things have gradually improved, some nurses still carry the weight of what the pandemic left behind. Although the percentage of nurses reporting severe symptoms of depression is low (3.1%), this indicates that some individuals may experience prolonged psychological effects even after the crisis has ended. Some are still finding their way through the pain, and may require time and ongoing professional support, highlighting the need for personalized and structured interventions for psychological rehabilitation.

The fact that 3.1% still face extremely severe anxiety after the pandemic shows just how deeply traumatic this experience has been for some. The findings emphasize that psychological consequences should not only be assessed statistically but also by taking into account the individual and professional context of nurses. Compared to the national and regional literature, the study reports that the rates of depression and anxiety are lower. Previous studies in Albania, Kosovo, and beyond during the peak of the pandemic reported higher levels of psychological symptoms [15,16,17]. This difference may reflect a better recovery of Albanian nurses in the post-pandemic period, perhaps due to experience gained, training received or coping strategies developed during the pandemic, compared with a meta-analysis that included 93 studies with 93,112 nurses, where the prevalence of depression was 35%, anxiety 37%, and stress 43% [3]. When comparing our results with the study conducted in Egypt, where 61.8% of nurses reported depressive symptoms [18], our findings appear more encouraging. Similarly, an umbrella review, which included 44 meta-analyses, evidenced a high prevalence of anxiety (29.9%), depression (28.4%), and sleep disorders (36.9–42.0%) among hospital workers [19]. After the pandemic, nurses in this study reported higher levels of emotional, social, spiritual, and environmental well-being; while physical and financial dimensions remained, lower [9], which is something that really resonates particularly with what we have seen. Nurses who felt they were emotionally recovering or even growing through the experience showed fewer signs of burnout and depression. This suggests that interventions aimed at improving institutional support and facilitating emotional processing of traumatic experiences may positively impact the recovery of emotional well-being in nurses. The difficulties in physical and financial recovery are understandable; chronic fatigue, long hours, and lack of vacations have negatively affected physical health, while economic uncertainty has increased stress and emotional consequences. The study by McBride et al. found that 78% of healthcare workers in the UK had ongoing financial concerns, with nurses at higher risk of depression [20]. Both the report by McGloin in Occupational Medicine [21] and the qualitative study by Maghsoodi et al. [22] reflect similar trends reported in this study: they shed light on just how deeply the pandemic has affected the emotional lives of healthcare professionals.

The experience during the pandemic influenced not only the increase in stress but also the personal and professional growth of nurses. Alquwez et al. identified four main spiritual groups [10]. Rogers et al. confirmed the increase in sense of purpose and professional fulfillment during the pandemic [8], while the meta-synthesis of Liu et al. emphasized the development of self-regulation and post-traumatic growth [7]. Our findings confirmed a statistically significant negative association between depression, anxiety, and stress with all eight dimensions of well-being. The data showed an inverse relationship between emotional, physical and social well-being and levels of psychological distress.

To further strengthen the interpretation of the results and identify the factors most influential on nurses’ mental health after the pandemic, ordinal logistic regression was applied. This multivariate analysis revealed that age, work experience, and involvement in COVID-19 wards were significant predictors of higher levels of depression and anxiety. Older nurses and those with more than 10 years of experience were more likely to experience psychological distress, showing a statistically significant association between long experience and increased levels of psychological distress.

Additionally, working in COVID-19 wards was associated with increased distress, possibly due to prolonged exposure to stressful conditions and ongoing risks during the pandemic. On the other hand, gender and training did not have a statistically significant impact, suggesting that experience and professional context play a greater role in shaping mental well-being. These findings underscore the importance of implementing supportive policies that take into account age, knowledge, and work experience in high-stress situations.

The study by Yu et al. supports this approach, noting that social support and a sense of purpose at work are protective factors against symptoms of depression and anxiety [6]. Kohnen et al. emphasize that emotional and spiritual well-being improves psychological resilience [23]. In our study, we also observed a close link between financial well-being and mental health, a connection similarly highlighted by McBride et al. [20]. When nurses feel financially secure, it eases some of the emotional weight they carry.

Binsaeed et al. also brought attention to something we saw in our findings: nurses who lived with a fear of COVID-19 and constant stress experienced a noticeable decline in their mental well-being [4]. In this light, our results strongly support the need for a holistic approach. It is not enough to alleviate symptoms; comprehensive interventions that address all dimensions of well-being, physical, social, and spiritual, are needed to support nurses’ long-term recovery and improvement. It is important to focus on the essential aspects that affect their individual well-being, including building functional social support, creating real opportunities for professional development, and improving their workplace experience. All of these are essential, not optional, for their well-being and the future of healthcare. Our finding supports a holistic approach to improving nurses’ mental health, one that goes beyond treating symptoms and focuses on strengthening every aspect of their well-being. This means standing beside nurses in real, everyday ways; being there with a social support they can rely on, creating space for them to grow professionally, creating safe and sustainable working conditions that support the well-being of nursing staff, and not forgetting that their emotional and spiritual well-being matters as much as their clinical skills when we take these elements seriously tosustain the long-term strength and dedication of the nursing workforce.

This study fills an important research gap in the Albanian context, providing data on the long-term impacts of the COVID-19 pandemic on the mental health and well-being of nurses. Using internationally validated instruments (DASS-21 and the 8-Dimensional Well-being Assessment),our findings are consistent with the global literature and provide a valuable cultural perspective. This adds value to nursing science in the region and can serve as a starting point for tailored interventions in health systems in transition. This study contributes theoretically by integrating the eight dimensions of the well-being model into a previously unexplored cultural context, such as Albania, thereby providing an extension of the international application of this framework. Practically, it provides a database for building personalized interventions in healthcare institutions to support the recovery and well-being of nurses in the post-pandemic period.

### Limitation of the Study

This study has several limitations that should be considered when interpreting the results. One important aspect is that the data were collected through self-report questionnaires, which may have influenced the accuracy of responses either because participants may not have entirely recalled specific experiences or because they may have felt inclined to respond in a way that seems more socially acceptable. Second, the selection of a non-nationally representative sample limits how well the findings can reflect the experiences of the wider nursing population. The use of purposive sampling and the selection of participants based on availability may have introduced selection bias, potentially affecting the balance of demographic and professional characteristics of the sample. Furthermore, as this is a cross-sectional descriptive study, the results reflect only correlation associations and do not allow for causal interpretations. Finally, the data for the pandemic period are retrospective, which may affect objective comparison with the post-pandemic period.

Future research should employ longitudinal designs and larger samples to assess the long-term impact of the pandemic. To mitigate these limitations in future research, it is recommended that triangulation methods (combining different data sources), random participant selection, and the use of longitudinal designs be employed to enable a more accurate, objective, and consistent assessment of nurses’ mental well-being.

The use of combined methods and the evaluation of structured support interventions may provide more profound practical insights into improving nurses’ well-being.

Despite its limitations, this study provides valuable insights into the long-term impact of the COVID-19 pandemic on nurses’ mental health and well-being. The use of valid instruments such as the DASS-21 and the Personal Assessment: 8 Dimensions of Well-being questionnaire strengthens the reliability of the data collected. Additionally, capturing information from both during and after the pandemic offers a broader perspective on how nurses’ experiences and wellness have evolved over time. Although the study provides new insights into nurses’ well-being after the pandemic in Albania, the results should be interpreted with caution due to the descriptive design and regional sample. We recommend that future research have a longitudinal design and nationally representative samples to verify and extend these findings. Although the study was conducted in two regional hospitals (Fier and Vlora), the limited number of sites may still restrict the broader applicability of the findings within the national healthcare context.

## 5. Conclusions

This study sheds light on the profound and ongoing impact that the COVID-19 pandemic has had on the mental health and well-being of nurses, an effect that persists even though the pandemic has been declared over. However, the fact that some nurses continue to struggle with severe symptoms highlights the ongoing need for thoughtful, structured, and compassionate support to help them fully heal and thrive. The assessment using the eight dimensions of well-being showed that the emotional, social, and spiritual dimensions were more developed, while the physical and financial ones remained fragile. This indicates that while the social and emotional aspects can be recovered more quickly through experience and human interaction, fatigue and economic insecurity require sustained institutional and systemic interventions.

One of the most meaningful findings of this study was the clear and significant link between emotional distress and lower levels of well-being in every area of life. This reminds us how essential it is to care for nurses in a truly holistic way, going beyond the treatment of symptoms to also nurture their inner balance, personal resilience, and professional fulfillment. In particular, spiritual and professional dimensions emerged as important supporting factors, helping nurses to better cope with challenges and strengthening their sense of purpose in their professions. When nurses manage to thrive both personally and professionally during difficult times, it makes us build an organizational culture that not only values high performance but also genuinely cares about the overall well-being of nurses. To achieve this, it is recommended that health institutions invest in implementing integrated mental health programs in the workplace, which include psychological support, reflective sessions, and professional mentoring. Policies that strengthen professional development and ensure fair remuneration are also necessary, as well as concrete interventions to improve the physical and financial dimensions of nurses’ well-being. These interventions should be sustainable and institutionalized so that well-being becomes an integral part of the assessment and support of human resources in the health system.

## Figures and Tables

**Table 1:** Distribution of demographic and occupational characteristics.

Table No. 1: 1Demographic and Occupational Characteristics		N	(%)
Gender	Male	52	17.9%
	Female	236	82.1%
Work Experience	≤5 years	74	25.7%
	6–10 years	82	28.5%
	11–20 years	57	19.8%
	>20 years	75	26.0%
Department where you worked during COVID-19	Intensive Care Unit (ICU)	11	3.8%
	Surgery	22	7.6%
	Operating Room	12	4.2%
	ENT/Ophthalmology	15	5.2%
	Emergency	38	13.2%
	Pathology	24	8.3%
	COVID-19 Dedicated Ward	55	19.1%
	Pediatrics	41	14.2%
	Dispensary	4	1.4%
	Maternity	52	18.1%
	Pediatrics/ICU	14	4.9%
Current Department	Intensive Care Unit (ICU)	12	4.2%
	Surgery	14	4.9%
	Infectious Diseases	26	9.0%
	Operating Room	15	5.2%
	ENT/Ophthalmology	16	5.6%
	Emergency	32	11.1%
	Pathology	44	15.3%
	Pediatrics	45	15.6%
	Dispensary	2	0.7%
	Maternity	63	21.9%
	Imaging	2	0.7%
	Pediatrics/ICU	17	5.9%
Average working hours per week during COVID-19	<40 h	38	13.2%
	40 h	152	52.8%
	>40 h	98	34.0%
Current weekly working hours	<40 h	39	13.5%
	occupational 40 h	173	60.1%
	>40 h	76	26.4%
Trained during the COVID-19 period	Yes	253	87.8%
	No	35	12.2%

**Table 2:** Distribution of numerical variables (age, DASS during and after COVID-19, 8 Dimensions of Well-being).

Variables		Nurses (*n* = 288)							
		Mean	±SD	Median	Mode	Minimum	Maximum	Qp	Qs
Age		38.56	10.42	38	40	22	62	30	46
During the COVID-19	Depression	6.22	4.34	6	6	0	21	3	8
	Anxiety	6.11	4.8	5	4	0	21	3	8
	Stress	8.3	3.6	8	10	0	18	6	10
After the COVID-19	Depression	3.26	5.18	2	0	0	21	0	4
	Anxiety	2.91	5.17	0	0	0	21	0	4
	Stress	4.75	5.3	4	0	0	21	0	8
8 Dimensions of Wellness	Emotional	32.41	6.44	34	36	10	40	30	37
	Spiritual	31.89	6.55	34	35	10	40	30	36
	Physical	30.79	5.77	32	33	10	40	28.25	34.75
	Social	32.32	6.48	33	40	10	40	30	37
	Financial	31.36	6.56	33	34	10	40	30	35
	Career	31.83	6.68	33	40	10	40	30	36
	Intellectual	32.17	6.92	34	40	10	40	30	37
	Environmental	32.18	6.92	33	40	10	40	30	37

**Table 3:** Cut-off scores for conventional severity labels (normal, moderate, severe).

	Depression	Anxiety	Stress
Normal	0–9	0–7	0–14
Mild	10–13	8–9	15–18
Moderate	14–20	10–14	19–25
Severe	21–27	15–19	26–33
Extremely Severe	28+	20+	34+

**Table 4:** Distribution of severity of depression, anxiety, and stress among nurses during and after the pandemic, expressed in frequency and percentage.

		During the COVID-19	After the COVID-19
Depression	Normal	243 (84.4%)	251 (87.2%)
	Mild	23 (8%)	16 (5.6%)
	Moderate	13 (4.5%)	11 (3.8%)
	Severe	10 (3.5%)	9 (3.1%)
Anxiety	Normal	196 (68.1%)	241 (83.7%)
	Mild	34 (11.8%)	9 (3.1%)
	Moderate	44 (15.3%)	25 (8.7%)
	Severe	5 (1.7%)	3 (1.0%)
	Extremely Severe	10 (3.5%)	9 (3.1%)
Stress	Normal	271 (94.1%)	275 (95.5%)
	Mild	17 (5.9%)	3 (1.0%)
	Moderate	10 (3.5%)	0 (0.0%)

**Table 5:** Results of Kendall’s Tau correlation between depression, anxiety, stress (after COVID-19), and the 8 Dimensions of Well-being.

Kendall’s Tau correlation			Depression after COVID-19	Anxiety after COVID-19	Stress after COVID-19	No. of Cases
8 Dimensions of Well-being	Emotional	r	−0.299	−0.298	−0.279	288
		*p*-value	<0.001 *	<0.001 *	<0.001 *	288
	Spiritual	r	−0.337	−0.379	−0.304	288
		*p*-value	<0.001 *	<0.001 *	<0.001 *	288
	Physical	r	−0.167	−0.267	−0.124	288
		*p*-value	<0.001 *	<0.001 *	0.005 *	288
	Social	r	−0.34	−0.352	−0.33	288
		*p*-value	<0.001 *	<0.001 *	<0.001 *	288
	Financial	r	−0.172	−0.249	−0.142	288
		*p*-value	<0.001 *	<0.001 *	0.001 *	288
	Career	r	−0.263	−0.265	−0.225	288
		*p*-value	<0.001 *	<0.001 *	<0.001 *	288
	Intellectual	r	−0.332	−0.308	−0.309	288
		*p*-value	<0.001 *	<0.001 *	<0.001 *	288
	Environmental	r	−0.332	−0.311	−0.287	288
		*p*-value	<0.001 *	<0.001 *	<0.001 *	288

* Significant *p*-value < 0.05. *p*-values are highlighted in light blue to emphasize statistically significant results (*p* < 0.05). All values marked with an asterisk (*) represent statistically significant correlations.

**Table 6:** Ordinal logistic regression on the impact of demographic and occupational factors on the level of depression after the pandemic.

Variables	B	SE	Wald	df	*p*-Value	OR (Exp(B))	95% CI for OR
Age (Mosha)	0.084	0.036	5.610	1	0.018	1.088	[1.015, 1.167]
Gender (Gjini = Female)	−0.606	0.500	1.467	1	0.226	0.546	[0.205, 1.454]
COVID-19 Training (Yes)	−0.185	0.660	0.078	1	0.780	0.831	[0.228, 3.033]
Department (COVID Unit)	0.984	0.424	5.398	1	0.020	0.374	[0.163, 0.857]
Experience (>10 years)	2.810	1.141	6.067	1	0.014	16.607	[1.775, 155.336]

*p*-values are highlighted in light blue to emphasize statistically significant results (*p* < 0.05).

**Table 7:** Ordinal logistic regression for anxiety after the COVID-19 pandemic.

Variables	B	SE	Wald	*p*-Value	OR (Exp(B))	95% CI për OR
Age	0.075	0.033	5.045	0.025	1.078	1.01–1.15
Gender = Male	−0.953	0.426	5.009	0.025	0.385	0.167–0.888
Trained during COVID = Yes	−0.754	0.645	1.365	0.243	0.47	0.133–1.667
Work Experience = 1	0.945	0.847	1.246	0.264	2.573	0.489–13.529
Work Experience = 2	0.708	0.671	1.113	0.291	2.029	0.545–7.554
Work Experience = 3	2.313	0.862	7.209	0.007	10.11	1.867–54.712
Department = COVID 19	−1.429	0.474	9.082	0.003	0.24	0.094–0.616

## Data Availability

The authors will make the raw data supporting this article’s conclusions available upon request.

## Abbreviations

The following abbreviations are used in this manuscript:

COVID-19	Corona virus Disease 2019
DASS-21	Depression, Anxiety and Stress Scale—21 items
ICU	Intensive Care Unit
